# 3D reconstruction of genomic regions from sparse interaction data

**DOI:** 10.1093/nargab/lqab017

**Published:** 2021-03-22

**Authors:** Julen Mendieta-Esteban, Marco Di Stefano, David Castillo, Irene Farabella, Marc A Marti-Renom

**Affiliations:** CNAG-CRG, Centre for Genomic Regulation (CRG), Barcelona Institute of Science and Technology (BIST), 08028 Barcelona, Spain; CNAG-CRG, Centre for Genomic Regulation (CRG), Barcelona Institute of Science and Technology (BIST), 08028 Barcelona, Spain; CNAG-CRG, Centre for Genomic Regulation (CRG), Barcelona Institute of Science and Technology (BIST), 08028 Barcelona, Spain; CNAG-CRG, Centre for Genomic Regulation (CRG), Barcelona Institute of Science and Technology (BIST), 08028 Barcelona, Spain; CNAG-CRG, Centre for Genomic Regulation (CRG), Barcelona Institute of Science and Technology (BIST), 08028 Barcelona, Spain; Centre for Genomic Regulation (CRG), Barcelona Institute of Science and Technology (BIST), 08003 Barcelona, Spain; Universitat Pompeu Fabra (UPF), 08002 Barcelona, Spain; ICREA, 08010 Barcelona, Spain

## Abstract

Chromosome conformation capture (3C) technologies measure the interaction frequency between pairs of chromatin regions within the nucleus in a cell or a population of cells. Some of these 3C technologies retrieve interactions involving non-contiguous sets of loci, resulting in sparse interaction matrices. One of such 3C technologies is Promoter Capture Hi-C (pcHi-C) that is tailored to probe only interactions involving gene promoters. As such, pcHi-C provides sparse interaction matrices that are suitable to characterize short- and long-range enhancer–promoter interactions. Here, we introduce a new method to reconstruct the chromatin structural (3D) organization from sparse 3C-based datasets such as pcHi-C. Our method allows for data normalization, detection of significant interactions and reconstruction of the full 3D organization of the genomic region despite of the data sparseness. Specifically, it builds, with as low as the 2–3% of the data from the matrix, reliable 3D models of similar accuracy of those based on dense interaction matrices. Furthermore, the method is sensitive enough to detect cell-type-specific 3D organizational features such as the formation of different networks of active gene communities.

## INTRODUCTION

Chromatin within the nucleus is organized into higher order structures that emerge at different genomic scales, from chromosome territories (at tens of megabases scale), active and inactive chromatin domains (at few megabases scale) (1), self-interacting domains or TADs (at hundreds of kilobases scale) (2,3,4) and long-range chromatin loops between regulatory elements (at tens of kilobases scale). This multi-scale organization has a direct impact on many biological processes, such as gene regulation, DNA replication and cell differentiation (5,6,7). Indeed, genome structure typically reflects cell-type-specific differences in the transcription pattern, and it is frequently rewired upon cell state changes and disease onset (8). Thus, investigating the principles shaping chromosome three-dimensional (3D) structure is pivotal to shed light into the relationship between genome structure and function.

Several experimental techniques are available to examine chromatin organization (9). Amongst them, molecular biology methods, such as chromosome conformation capture (3C) and its derivatives are widely used (10). These experiments retrieve information about the frequency of interaction between loci in single (11,12,13) or in populations of thousands to millions of cells and have been designed to analyse the chromatin landscape at different genomic scales (1,14,15,16). For example, some cell population-based experiments allow the retrieval of unspecified interactions in the whole genome (e.g. Hi-C (1), Micro-C (14), GAM (15) and SPRITE (16)). Complementarily, other 3C-based experiments are tailored to capture interactions centred on a specific locus with the rest of the genome (e.g. 4C (17) and multi-contact 4C (MC-4C) (18)) or on sets of dispersed loci in the genome, such as loci enriched for a specific protein (HiChIP) (19) or loci harbouring gene promoters (pcHi-C) (20). Each class of 3C-based experiments provide different but complementary insights on particular aspects of the genome organization, and their analysis is dependent on the experimental genomic resolution and on the inherent technical biases of each experimental procedures.

A variety of physics- and data-driven approaches for genome 3D reconstruction have been developed to expose the principles shaping chromosome 3D structure (21,22,23,24). For instance, data-driven (restraint-based) modelling approaches as PGS (25,26), TADbit (27), 4Cin (28) and TADdyn (29) have been implemented to reconstruct ensembles of chromatin 3D models from cell population-based datasets. Others are focused on the 3D modelling of chromatin based on single-cell Hi-C data, like manifold based optimization (30) and NucDynamics (31). However, the majority of the data-driven methods are based on interaction experiments that have been designed to retrieve dense contact information from a continuous set of loci or the whole genome, whilst other interaction experiments are characterized by data sparseness (e.g. HiChIP or pcHi-C). As such, data-driven methods for sparse data modelling are needed.

Generally, the interaction profiles of sparse 3C-based datasets have specific properties that set them apart from other 3C-like techniques characterized by a dense interaction profile. Indeed, protein or promoter capture-based interaction profiles are heavily biased on interactions between captured fragments and devoid of interactions between non-captured fragments. This fact poses the question of whether this lack of information prevents the 3D reconstruction of the whole loci of interest and its analysis, or whether it is sufficient to allow for accurate 3D modelling. To answer this question, we have implemented a new method, which is tailored to integrative modelling and analysis of sparse 3C-based datasets. We have also validated the procedure comparing the resulting reconstructed models with available dense experimental datasets, unveiling that the 3D chromatin organization can be well recovered by interrogating only a small percentage of loci. Additionally, we have designed new tools to facilitate a robust differential analysis of the resulting models and showcased their usability in comparative analyses using the β-globin locus as a test case. Interestingly, comparing different cell-types, we unveiled that the β-globin locus in cord-blood Erythroblasts (cb-Ery), where its foetal and adult β-globin genes are highly expressed, is hierarchically organized in a 3D network of active gene communities that follows an expression gradient.

## MATERIALS AND METHODS

### Experimental datasets

Structural data were obtained from publicly available 3C-based chromatin interaction experiments of GM12878 cells (Hi-C GEO: GSE63525 and pcHi-C ArrayExpress: E-MTAB-2323) (6,32), and cord-blood derived Erythroblasts (cb-Ery), naive CD4+ T-cells (nCD4), and Monocytes (Mon) (pcHi-C EGA: EGAS00001001911) (33).

#### Hi-C datasets processing

The reads for each replicate were mapped onto the GRCh38 reference genome, filtered and merged using TADbit with default parameters (27). Then, starting from the merged filtered fragments, the genome-wide raw interaction maps were binned at 5 kilo-base (kb) and normalized using OneD (34) as implemented in TADbit (27).

#### pcHi-C datasets processing

For each experiment, the reads were mapped onto the GRCh38 reference genome using TADbit (27) and were filtered applying the following filters: (i) self-circles, (ii) dangling-ends, (iii) errors, (iv) extra dangling-ends, (v) duplicated reads and (vi) random breaks. Next, we computed the reproducibility score to measure the similarity between replicates from each pcHi-C dataset (35). Then, for each cell-type, the different replicates from the same experiment were merged into one dataset for further analysis, making an exception with replicate ERR436029 from the GM12878 pcHi-C dataset (E-MTAB-2323), which was discarded due to a clearly low reproducibility score when compared with the rest of the replicates (average of 0.24 with the other replicates as compared to the average of 0.84 obtained between the other replicates). Using the merged filtered fragments, the genome-wide raw interaction maps of each cell-type were binned at 5 kb and normalized using the PRoportion of INTeraction approach (PRINT, next section).

#### Sparse data normalization PRoportion of INTeraction approach (PRINT)

PRINT, a multi-stage normalization procedure, weighs each pair of interacting bins with the same philosophy as the visibility approach for Hi-C (36). Starting from a raw interaction matrix as input, PRINT first transforms the raw interaction between two bins (*i* and *j*) into a percentage of interaction with respect to the rest of the genome as:

$$\begin{equation*}{\rm valu}{e_{ij}} = \frac{{{\rm bi{n}_{ij}}}}{{\sum {{\rm row}_i} + \sum {{\rm row}_j} - {{\rm bin}_{ij}}}} \end{equation*}$$

where (bin*_ij_*) represent the number of times in which bin *i* and *j* interact, and ∑row*_i_* and ∑row*_j_* are the sum of all the interactions of bins *i* and *j*, respectively, with all the genome (self-interactions included). Then, the non-baited interactions (that is, those bins containing only pcHi-C off-target reads) are filtered out.

#### PRINT assessment

Using the *benchmarking datasets* described above, each stage of PRINT normalization (raw pcHi-C (pcHi-C-raw), pre-normalized pcHi-C (pcHi-C-pre) and normalized pcHi-C (pcHi-C-norm)) was assessed in comparison with the dense Hi-C interaction matrix by calculating the Spearman's rank correlation coefficient between interactions (bin*_ij_)* present in both interaction matrices.

### Reconstructed 3D genomic regions

#### Benchmarking datasets

We selected 12 genomic regions of interest (Supplementary Table S1) as defined by Rao *et al.* (6). This set of genomic regions were predicted to result in reliable 3D models based on their >0.7 MMP scores (37) (Supplementary Table S2). Briefly, MMP score takes into account the interaction matrix size, the contribution of significant eigenvectors in the matrix and the skewness and kurtosis of the *z*-scores distribution of the matrix to assess their potential for being modelled (37).

#### Comparative analysis datasets

We selected a genomic region around a locus of interest (here the β-globin) defining it in a semi-automatic manner in each cell-type. Briefly, a viewpoint, which may be constituted by a bin or a set of bins of interest, is selected. Here, as viewpoint we used bins enclosing the active haemoglobin genes in cb-Ery (HBB, HBD, HBG1 and HBG2). Then, all the other bins that interacted with the viewpoint bins in the normalized genome-wide interaction matrix were selected. Each of these bins were then scored by their cumulative normalized interaction frequency values with the viewpoint bins. From this set only the top intra-chromosomal 200 bins were selected since, by visual inspection, they were the bins spanning the genomic region that best enclosed the viewpoint. Then an unweighted interaction network was generated with the nodes corresponding to the top 200 bins and the viewpoint bins. Edges between nodes were added if their pairwise cumulative normalized interaction frequency value was in the top 200 interacting bins. Then, a series of transformations were applied to the unweighted interaction network: (i) nodes that are highly proximal in 1D genomic resolution (closer than 25 kb) were merged into one node; and (ii) poorly connected nodes in the network that had <5 edges were filtered out (average number of edges per node in Mon, nCD4 and cb-Ery were 200, 214 and 214, respectively). The extreme nodes in terms of genomic coordinates were selected from the final unweighted interaction network to represent the optimal genomic region around the viewpoint. Here, to perform comparative analysis, we defined the optimal genomic region around the viewpoint as the broader genomic region that enclosed all of the genomic coordinates identified in each cell-type.

### 3D chromosome ensemble reconstruction from sparse datasets

#### Model representation

Each genomic region was described with a beads-on-string model based-on the previously implemented protocols (29,38) without bending rigidity potential. Thus, a chromosome was represented with *N* spherical beads with diameter *σ* = 50 nm that contain 5 kb of chromatin which determined the genomic unit length of each model.

#### System set up for molecular dynamics

All simulations were done using TADdyn (29). A generic random self-avoiding walk algorithm was used to define the initial conformation of each model. The potential energy of each system comprised the terms of the Kremer–Grest polymer model (39) including chain-connectivity (Finitely Extensible Nonlinear Elastic, FENE) (40) and excluded volume (purely repulsive Lennard-Jones) interactions. The initial conformation was placed randomly inside a cubic simulation box of size 1000 σ centred at the origin of the Cartesian axis O = (0.0, 0.0, 0.0), tethered at the centre of the box using a harmonic (*K*_t_ = 50.0 k_B_T/${{\rm{\sigma }}^2}$ and d_eq_ = 0.0 σ) to avoid any border effect and energy minimized using a short run of the Polak–Ribiere version of the conjugate gradient algorithm (41) to favour smooth adaptations of the implementations of the excluded volume and chain connectivity interaction.

#### Encoding sparse data into TADdyn restraints

TADdyn (29) empirically identifies the three optimal parameters to be used for modelling based on a grid search approach. These are: (i) maximal distance between two non-interacting particles (*maxdist*); (ii) a lower-bound cut-off to define particles that do not frequently interact (*lowfreq*); and (3) an upper-bound cut-off to define particles that frequently interact (*upfreq*). All possible combinations of the parameters were explored in the intervals *lowfreq* = (−1.0,−0.5, 0, 0.5), *upfreq* = (−1, −0.5, 0, 0.5), *maxdist* = (200, 300, 400, 500) nm and assessing each combination using distance thresholds to determine if two particles are in contact (*dcutoff*) at 100,150, 200, 250, 300, 350, 450, 500 nm. For each of the combinations an ensemble of 100 3D models was generated and the Spearman correlation coefficient between the contact map derived from each ensemble and the experimental input interaction matrix was calculated. The top set of parameters for each region in each cell-type were set for those resulting in the highest Spearman correlation coefficient between the models contact map and the input interaction matrix. To allow for a robust comparative analysis (‘Materials and Methods’ section) the optimal *maxdist* and the *dcutoff* parameters were selected based on the consensus within the top optimal values for each region in each cell-type. Optimal *maxdist* and the *dcutoff* were set at 300 and 200 nm, respectively for the ensembles of models reconstructed from the GM12878, cb-Ery, nCD4 and Mon pcHi-C datasets. Once the three optimal parameters were defined, the type of restraints between each pair of particles was set considering an inverse relationship between the frequencies of interactions of the contact map and the corresponding spatial distances. Non-consecutive particles with contact frequencies above the upper-bound cut-off were restrained by a harmonic oscillator at an equilibrium distance, whilst those below the lower-bound cut-off were maintained further apart than an equilibrium distance by a lower-bound harmonic oscillator. To identify 3D models that best satisfy all the imposed restraints, the optimization procedure was then performed using a steered molecular dynamic protocol. A total of 1000 replicate trajectories were generated for each genomic region and dataset. Each of the 1000 replicate trajectories, the conformation at the end of the steering protocol (when the target spring constant and equilibrium distance are reached) was retained to form the final ensemble of 1000 3D models. For the cb-Ery, nCD4 and Mon datasets, to account for possible mirrored 3D models within the final ensemble of 3D models, each ensemble was then clustered based on structural similarity score as implemented in TADbit (27) and only the models from the most populated cluster were retained for further analysis.

#### Steered molecular dynamics protocol

A steered molecular dynamics protocol was used to progressively favour the imposition of the defined set of restraints between non-consecutive particles. For each restraint, the equilibrium distance was set to 1 particle diameter (σ). The spring constant *k(L,t)* was weighted with the sequence-separation *L* between the constrained beads as in TADdyn (29) to ensure that the steering process was not dominated by the target pairs at the largest sequence separation. However, here the *k(L,t)* was smoothly ramped during the steering phase from zero to its maximum value.

### 3D chromosome ensemble reconstruction from dense datasets

The reconstruction of 3D models of genomic regions from dense data followed the modelling protocol described above. That is, a grid search approach was used to select for the optimal parameters to be used for modelling. The optimal *maxdist* and the *dcutoff* parameters were selected based on the consensus within the top optimal values for each region in the GM12878 pcHi-C dataset and set at 300 and 200 nm, respectively. Using these parameters, the final ensemble of 1000 3D models was obtained starting from the computed 1000 steered molecular dynamics trajectories.

### 3D chromosome ensemble reconstruction from Virtual pcHi-C derived from dense datasets

A dataset of Virtual pcHi-C interaction matrices was produced starting from the normalized Hi-C interaction matrices at 5 kb resolution (GM12878 cells GEO: GSE63525; ‘Materials and Methods’ section) and from the liftover (https://genome.ucsc.edu/cgi-bin/hgLiftOver) list of captured fragments in pcHi-C GM12878 experiment (32). The obtained Virtual pcHi-C interaction matrices comprised only interactions (bin*_ij_)* in which either *i* or *j* enclose the coordinates of a captured fragment. These interaction matrices were used as input for the reconstruction of 3D models of genomic regions following the modelling protocol described above. The optimal *maxdist* and the *dcutoff* parameters were set at 300 and 200 based on their consensus with the parameters used in the GM12878 pcHi-C dataset. A total of 1,000 steered molecular dynamics trajectories were computed, and for each trajectory the conformations satisfying the majority of the imposed constraints within a radius of 2 σ were retained.

### 3D chromosome ensemble reconstruction from ‘synthetic’ sparse dataset

We used a previously published ‘toy genome’ (37) (that is, the ensemble of models accounting for the formation of TAD-like architecture with low structural variability and high noise levels that comprises a total of 626 particles at the highest genomic resolution) to randomly select 10 sets of 22 loci from the toy genome contact map (or synthetic interaction maps). These loci mimic pcHi-C to generate reliable sparse interaction matrices comprising only interactions (bin*_ij_*) in which either *i* or *j* have been selected as random captured loci. Each of these sets was then randomly subsampled to generate ‘synthetic’ capture matrices with 2, 4, 6, 10, 14 and 18 selected captured loci. The obtained ‘synthetic’ capture matrices (70 in total) were next used as input for the reconstruction of 3D models of genomic regions following the modelling protocol described above. The optimal *maxdist* and the *dcutoff* parameters were set at 500 and 200 nm. Using these parameters, a final ensemble of 100 3D models was reconstructed for each ‘synthetic’ capture matrices comprising the conformations that best satisfied the imposed restraints in each of the computed 100 steered molecular dynamics trajectories.

### Analysis of the ensemble of 3D models

#### Contact map generation

For each ensemble of 3D models, a contact map was calculated at 5 kb resolution to visualize the frequencies of contacts in the ensemble. Two beads were considered to constitute a contact when their euclidean distance was below 200 nm cut-off.

#### Matrix comparison

The degree of similarity between two matrices was computed by comparing each cell from the matrices, or a subset of them, using the Spearman's rank correlation coefficient (*r_s_*) as implemented in the Python library SciPy (42,43):

$$\begin{equation*}{r_s} = 1 - \frac{{6\mathop \sum \nolimits_{i = 1}^n {{\left( {{r_{{\rm{bi}}{{\rm{n}}_{{x_i}}}}} - {r_{{\rm{bi}}{{\rm{n}}_{{y_i}}}}}} \right)}^2}}}{{n\left( {{n^2} - 1} \right)}}\end{equation*}$$

where ${r_{{\rm{bi}}{{\rm{n}}_{{x_i}}}}}$ is the rank of the *ith* observation in one matrix, ${r_{{\rm{bi}}{{\rm{n}}_{{y_i}}}}}$ is the rank of the *ith* observation in the other matrix and *n* states for the number of pairs of observations.

#### Particle-to-particle median distance correlation (ppMdC)

For each ensemble of 3D models, we differentiated three sets comprising particles enclosing the coordinates of: (i) captured loci (capture), (ii) non-captured loci (other) and (iii) all the loci (all). For each of the pairs of particles in a given set we calculated the particle-to-particle median distance. Then, the degree of similarity between two given sets was computed using the Spearman's rank correlation coefficient between their particle-to-particle median distances. The ppMdC measure varies between −1.0 and 1.0 for comparisons where the particle-to-particle median distances perfectly anti-correlate or correlate, respectively.

#### Hierarchical clustering of ensembles of 3D models

Multiple ensembles of 3D models were merged in a unique set and the models were structurally superpose using pairwise rigid-body superposition. Next, the all-vs-all distance root-mean-square deviation (dRMSD) was calculated and the resulting dRMSD matrix was hierarchically clustered using Ward's sum of squares method (44) as implemented in the Python library SciPy (42).

#### Cell-specific expression profile

Publicly available (33) expression matrix containing the expression values (log(FPKM)) of each gene in cb-Ery, nCD4 and Mon cell-types was downloaded (GeneExpressionMatrix.txt.gz at https://osf.io/u8tzp/). The three datasets had two or more replicates each (two cb-Ery, five Mac and eight nCD4, respectively), thus the average expression value of each gene from all replicates was used. Then, a cell-specific per-bin cumulative expression profile of the chr11:3 795 000–8 505 000 genomic region at 5 kb resolution was obtained assigning the mean expression value of each gene (with log(FPKM) > 0) to bins enclosing for the coordinates of its transcription start site (coordinates retrieved from bioMart (45)).

#### 3D enrichment analysis

To study the spatial co-localization of different regulatory elements and the local levels of transcription (based on genome-wide ChIP- and RNA-seq data) around a selected locus (central viewpoint) we implement a *3D enrichment analysis tool* (named ‘radial-plot’) that allows the comparison of heterogeneous sets of data from multiple data sources. Per each cell-type a per-particle binarized chromatin marks profile in the genomic region was generated starting from the ChIP-seq signal of H3K27ac, H3K36me3, H3K4me1, H3K4me3, H3K9me3 and H3K27me3 in cb-Ery, nCD4 and Mon cell-types (33). A particle was considered enclosing for a chromatin mark if a peak was present. Similarly, we also constructed, for each cell-type, a per-particle binarized transcription profile starting from the cell-specific expression profile (‘Materials and Methods’ section). Then the 3D spatial distribution of the 3D enrichment based on the per-particle binarized profile around the chosen central viewpoint was calculated as follow: (i) starting from the central viewpoint an initial sphere with a radius of 200 nm was constructed; (ii) a series of spherical shells, that occupied a volume equal the initial sphere, were added; (iii) each model in the ensemble of 3D models a particle of the binarized profile was assigned to a spherical shell based on its relative distance to the central viewpoint; (iv) each spherical shell we performed Fisher's exact tests for 2 × 2 contingency tables comparing the amount of particles with or without signal in the spherical shell with the outside ones, and the log of the odd ratios was assigned to the shell if the *P*-value < 0.01. The obtained 3D enrichment was then visualized as a 2D radial plot.

#### Defining gene communities: co-occurrence of expressed genes

For each ensemble of 3D models, based on their cell-specific expression profile (‘Materials and Methods’ section), we defined the set of expressed particles (log(FPKM) > 0). Then, considering this set of particles, an all-versus-all pairwise distances matrix was calculated in each model and hierarchically clustered using Ward's sum of squares method (44) as implemented in the Python library SciPy (42). Then the Calinski–Harabasz index (46), as implemented in the Python library Scikit-learn (47), was used to determinate the optimal number of clusters in each dendrogram. Then, for each ensemble, a co-occurrence matrix was generated considering the percentage of models in which a pair of particles belonged to the same cluster. The co-occurrence measure varies between 0 and 100, where 0 indicates absence of co-occurrence and 100 indicates a stable co-occurrence within the ensemble of 3D models. The co-occurrence matrix was next hierarchically clustered using Ward's sum of squares method (44) and communities of co-occurrent active genes were identified using the Calinski –Harabasz index analysis in the dendrogram.

#### Communities stability within the ensemble of models

To assess the stability of each community within the ensemble we introduced the inter-community co-occurrence score that defines the degree of unstable compositions of a community. It is computed as the mean co-occurrence values between each gene in a community and the rest of the communities.

#### Distance between communities and within community

To describe the spatial arrangement of each community for a given ensemble of 3D models, we treated each community as a rigid body and calculated its centre of mass (COM) in each 3D model of the ensemble. Per each model the all-versus-all pairwise distances between the COMs of each communities were computed and the mean distance values assigned as the typical distance between communities. Similarly, per each model, we also calculated the distance of each particle in a given community and the COM of its community. The within community distance of a given particle was defined by its mean value in the ensemble of 3D models.

## RESULTS

### Overall modelling strategy for sparse 3C data

**Figure 1.**
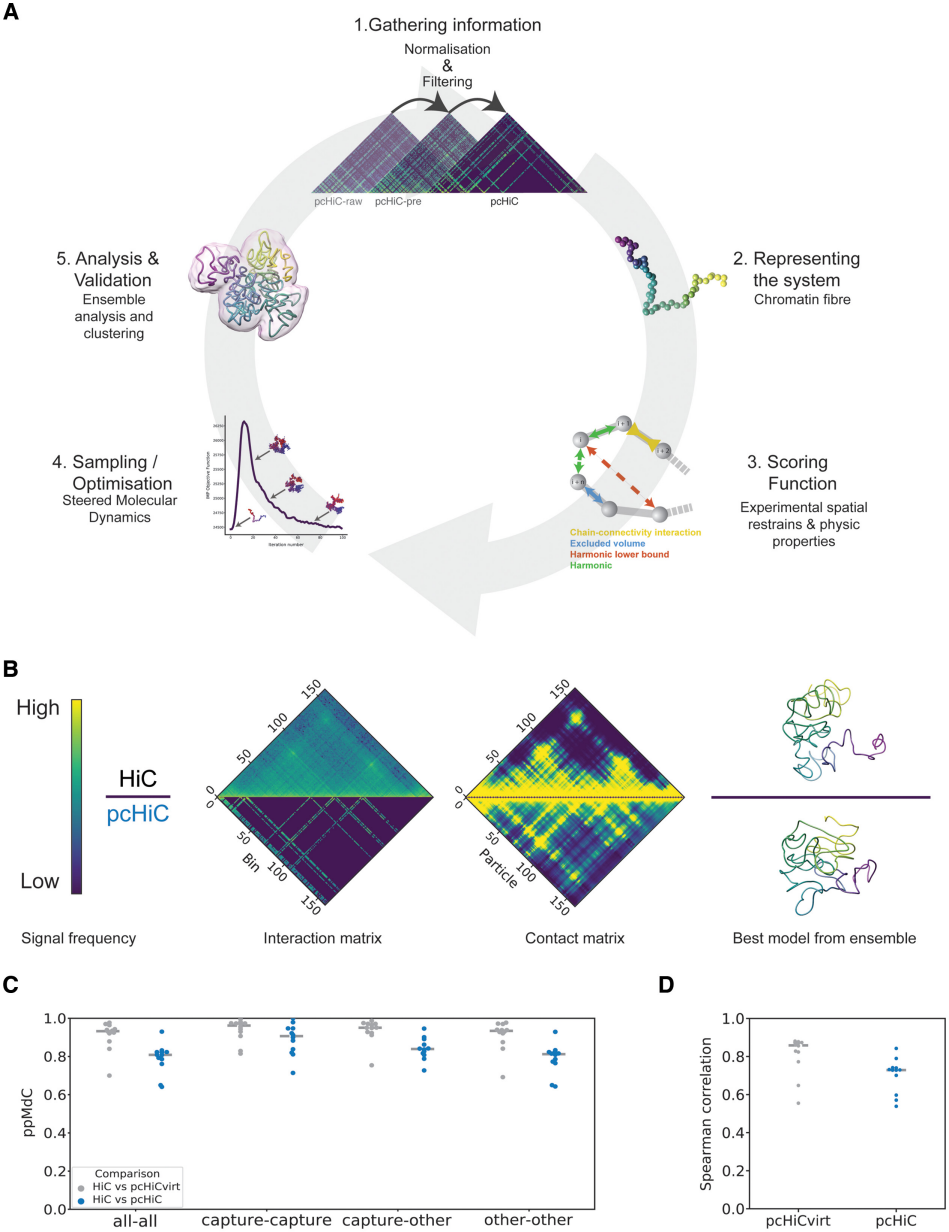
Integrative modelling for sparse datasets efficiently reconstructs the 3D organization of genomic loci. (**A**) Workflow of the integrative modelling approach followed to build ensembles of chromatin 3D models from pcHi-C: (i) gathering the input interaction matrices with subsequent normalization and filtering; (ii) representation of the chromatin fibre as a polymer with the particle size proportional to the resolution of the experiment; (iii) definition of the scoring function used in the modelling procedure. Here, the scoring function comprises spatial restraints derived directly from the input interaction data and from properties of the chromatin fibre (‘Materials and Methods’ section); (iv) sampling the conformational space by steered molecular dynamics (‘Materials and Methods’ section); and (v) validation of the obtained ensemble of models and further analysis. Model images in all panels were created with Chimera (74). (**B**) Representation of the input and output data from region 2 (Supplementary Table S1). The upper half of the panel refer to the dense dataset (Hi-C), whereas the lower half refer to the sparse-datasets (pcHi-C). From left to right, the matrices of normalized interaction frequency (‘Materials and Methods’ section) between each pair of bins, the contact matrix obtained from the ensemble of models of region 2 displays the percentage of models in which two bins are found bellow the defined distance cut-off for the contact (‘Materials and Methods’ section), and the best model from the ensemble as assesses by the scoring function. The colour bar shows the colour coding from low (blue) to high (yellow) interaction or contact frequencies signal. (**C**) Comparison between model ensembles derived from sparse (pcHi-Cvirt and pcHi-C in grey and blue, respectively) and dense (Hi-C) datasets assessed by the particle-to-particle median distance correlation (ppMdC; ‘Materials and Methods’ section). Three subsets of particles have been compared given the enclosed loci: (i) captured loci (capture), (ii) non-captured loci (other) and (iii) all the loci (all). The grey dashed line indicates the median ppMdC in the 12 analysed regions. (**D**) Element-wise Spearman correlation coefficients between the experimental Hi-C interaction matrices and the contact maps derived from the model ensembles reconstructed from sparse data (pcHi-Cvirt and pcHi-C in grey and blue, respectively). The grey dashed line indicates the median element-wise Spearman correlation coefficients in the 12 analysed regions.

Sparse 3C datasets provide information of interactions that involve a limited number of specific loci in the genome. pcHi-C, for example, provides a promoter-centred view of chromatin interactions, helping to assign distal regulatory regions to their target genes, thus providing insights on how gene expression might be controlled (32,33,48) and how disease-associated genomic variation could affect gene regulation (49). The main limitation of these sparse technologies, however, is the scarcity of specialized tools for their analysis. Here, we have developed an integrative 3D modelling method capable of dealing with data sparsity, enabling the analysis and interpretation of pcHi-C data, and tested it on 12 distinct loci (Benchmarking datasets; ‘Materials and Methods’ section and Supplementary Table S1). Our method follows an integrative modelling procedure comprising five steps (50): (i) gather experimental data and process them to obtain the input interaction matrix for the modelling approach, (ii) represent the selected chromatin regions using a bead-spring polymer model with a particle size proportional to the genomic resolution of the experimental data, (iii) transform the frequency of interactions into spatial retrains, (iv) sample the conformational space by steered molecular dynamics and (v) analyse and validate the obtained ensemble of 3D models (‘Materials and Methods’ section and Figure 1A).

In this work, we gathered pcHi-C interaction data (‘Materials and Methods’ section), whose processing step is pivotal to minimize the experimental biases from the capture protocol. To this end, we designed a multi-stage normalization procedure named PRINT (‘Materials and Methods’ section). PRINT weighs each interaction by dividing it by the cumulative whole-genome interaction frequencies of both of the interacting bins, regularizing the interaction patterns for the fact that captured loci are highly enriched in contacts. It also removes the pcHi-C unspecific interactions between non-probed bins. To test quantitatively the performance of our normalization procedure, we compared each of the normalization stages of the pcHi-C matrices with the respective Hi-C matrices normalized with OneD in each of the selected loci (34). The median correlation between bins with interaction data in both matrices was 0.27 (±0.025 Median Absolute Deviation (MAD)) for raw pcHi-C matrices (pcHi-C-raw), increasing to 0.44 (±0.032 MAD) with the pcHi-C pre-normalization step (pcHi-C-pre) and reaching 0.60 (± 0.056 MAD) for fully normalized pcHi-C matrices (pcHi-C-norm) (Supplementary Figure S1A), suggesting that PRINT reduced successfully the target biases. Then, we represented the selected loci as a bead-spring polymer model with a particle size set to 5 kb, taking into account the restriction fragment lengths distribution in the benchmarking datasets (Supplementary Figure S1B). Similarly to TADbit (27) and TADdyn (29), to simulate the structural conformation of genomic loci, we then transformed the interaction frequencies associated with each bin pair into spatial restraints (‘Materials and Methods’ section). The latter were then imposed on the model using steered molecular dynamics as sampling method in which the spring constant associated to each restraint was ramped up as a function of simulation time from zero to the value computed from the interaction data. Lastly, we implemented new means for a robust quantitative spatial differential analysis of genomic loci.

### Comparison between sparse and dense 3C-derived models

Dense 3C data have been extensively used to reconstruct the 3D organization of genomic loci (25,27,29,30). Here, to test the reliability of our modelling approach, we used sparse and dense datasets to build ensembles of models of the same loci. Specifically, we applied our integrative method for sparse data modelling to previously published pcHi-C datasets of GM12878 cells (32) to reconstruct 3D model ensembles of 12 distinct loci (Figure 1B and Supplementary Table S1) at a 5 kb resolution and compared them with the corresponding ones reconstructed using Hi-C (6) at the same genomic resolution. Additionally, to quantify the effect of sparsity in the comparison independently of the experimental protocol biases, we generated virtual pcHi-C (pcHi-Cvirt) interaction matrices from the normalized Hi-C datasets extracting the rows and columns probed in the pcHi-C experiment (‘Materials and Methods’ section). These virtual sparse matrices were then used to reconstruct 3D model ensembles of the selected loci.

The comparison between the sparse and dense derived 3D model ensembles revealed that it is possible to recover most of the 3D organization of the dense dataset in spite of the data sparsity (Figure 1C). Indeed, the all-versus-all particle-to-particle median distance correlation (ppMdC) between the sparse and dense derived 3D model ensembles was 0.81 (±0.019 MAD) and 0.93 (±0.024 MAD) for both pcHi-C and pcHi-Cvirt. Additionally, when comparing distances between particles that have both been captured in the pcHi-C experiment (capture–capture), the ppMdC was higher, reaching 0.91 (±0.054 MAD) for pcHi-C and 0.96 (±0.019 MAD) for pcHi-Cvirt. Consistently, when comparing distances between non-captured particles with captured particles (capture-other) or between non-captured particles (other–other), the ppMdC indicated good agreement with values of 0.84 (±0.03 MAD) and 0.95 (±0.02 MAD), and 0.81 (±0.02 MAD) and 0.93 (±0.02 MAD), respectively, for pcHi-C and pcHi-Cvirt in both comparisons (Figure 1C). The results indicate that the sparse derived ensembles of 3D models are a good representation of the dense experiment and that the intrinsic experimental biases of the capture experiment only minorly affect the 3D reconstruction. Indeed, comparing the whole contact map computed from the 3D model ensembles derived from sparse data directly with the whole experimental Hi-C interaction matrices revealed that the reconstructed ensembles of models are in good agreement with the dense experimental data having an element-wise Spearman's rank correlation coefficient of 0.73 (±0.02 MAD) and 0.86 (±0.02 MAD), for pcHi-C and pcHi-Cvirt derived ensembles of models, respectively (Figure 1D). Overall, this suggest that the ensembles of models reconstructed by our approach represent well the 3D organization of the selected genomic regions and, more importantly, recover the spatial arrangements of loci that are not interrogated by the sparse experiment.

### Reconstruction efficiency and data sparsity

**Figure 2.**
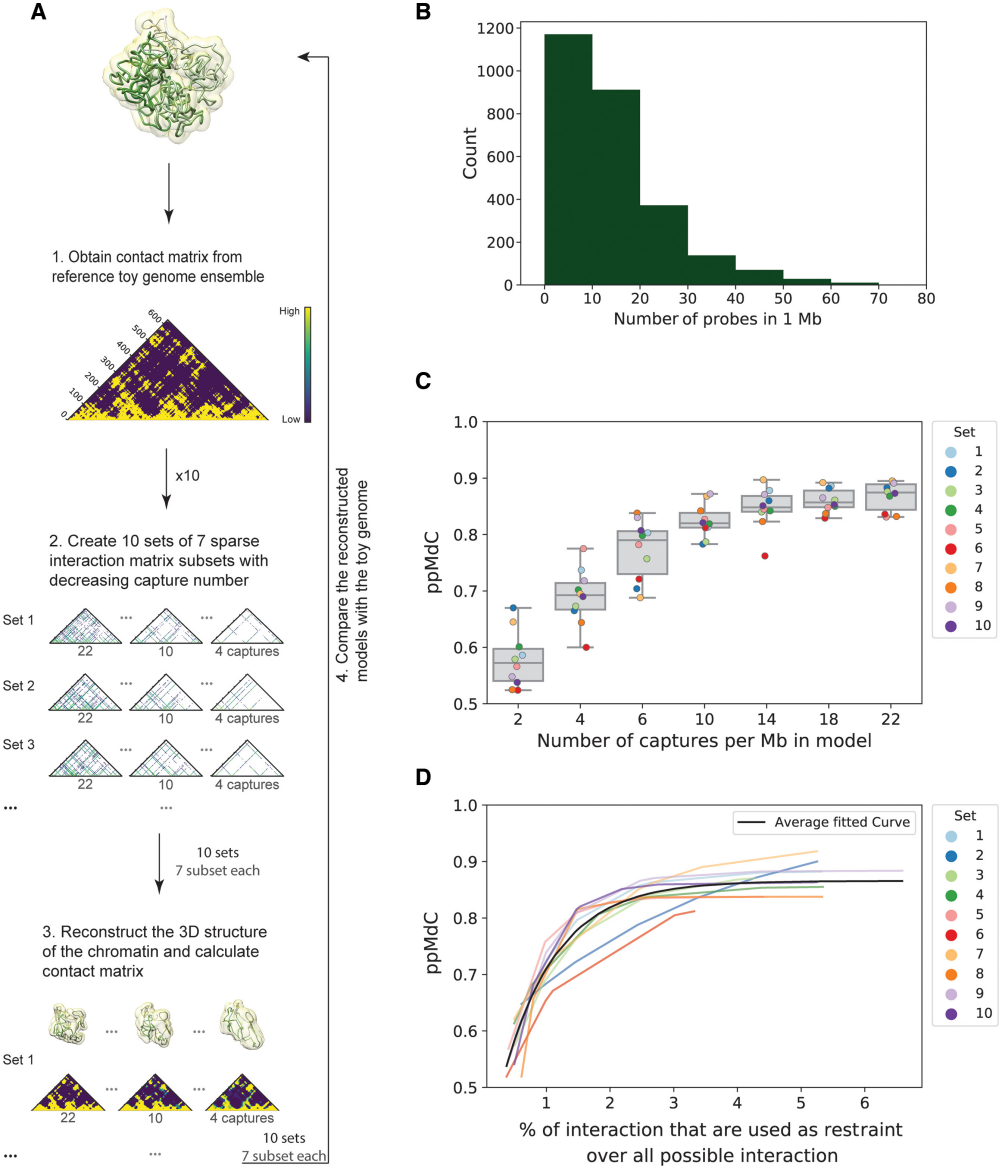
A low percentage of the interaction data is needed to produce reliable 3D reconstructions. (**A**) Workflow for the generation of 3D model ensembles from ‘synthetic’ sparse datasets and comparison with the toy genome. A total of 70 ‘synthetic’ captured map were generated representing 10 different capture experiments with different level of data sparsity (‘Materials and Methods’ section). Model images were created with Chimera (74). (**B**) Distribution of pcHi-C probes per megabase windows in the genome (32). (**C**) Distribution of the ppMdC between the ‘synthetic’ models and the toy genome grouped by subsets of captures per megabase. Box boundaries represent first and third quartiles, middle line represents median and whiskers extend to 1.5 times the interquartile range. The 10 sets of captured positions are displayed with the colour code shown in the insert. (**D**) Relationship between the ppMdC and the percentage of cells in the matrix used as restraints in each set represented with an exponential fit. The used colour code is the same as in (C), the grey line represents the mean fit of all the datasets in analysis.

To investigate the relationship between the reconstruction efficiency and data sparsity, we simulated ‘synthetic’ capture data. Briefly, we generated 10 different sets of ‘synthetic’ capture matrices that represent generic capture-like experiments. We started from the contact matrix derived from a 3D toy-genome models ensemble that simulates roughly a one Mb length genome (comprising more than 600 particles) with a TAD-like architecture, a high level of interaction noise and low variability between models (37) (‘Materials and Methods’ section and Figure 2A). To build each of the 10 ‘synthetic’ sets, we randomly selected 22 captured loci and constructed 6 additional datasets of different sparsity downsampling each set considering 2, 4, 6, 10, 14 and 18 loci at a time, which mimics the distribution of captured probes per Mb present in a typical genome-wide pcHi-C experiment (Figure 2B). The constructed 70 capture-like matrices thus aim to represent typical pcHi-C experimental design. Using our integrative modelling method for sparse datasets, we reconstructed, from each of the ‘synthetic’ capture matrices in the dataset and their downsampled counterparts, ensembles of 100 models and compared them with the reference toy-genome ensemble (Figure 2A). Independently of the sets, the ppMdC between the sparse and dense model ensembles increased with the number of captured particles used in the modelling procedure reaching a median correlation between sets of 0.82 (+/- 0.02 MAD with just 10 captures per Mb (Figure 2C). Notably, also with 4 and 6 captures per Mb the ppMdC reached 0.69 (±0.04 MAD) and 0.79 (±0.05 MAD) for four and six captures, respectively, although with greater variation within sets. This suggests that with 10 captured loci per Mb the uncertainty in the input information is smaller, leading to more precisely reconstructed models. Nevertheless, it is possible to reconstruct good models also with fewer as four captured loci per Mb although with a higher degree of variability. To quantify the effect of data sparseness on model reconstruction, we next measured the amount of input information used during the modelling as the percentage of all possible interaction pairs in the contact matrix (dense data input) and then assessed it with the ppMdC. The results indicate that it was possible for the majority of the sets (8/10) to reliably reconstruct the reference toy genome (ppMdC > 0.8) with just 2–3% of all the interaction pairs in the contact matrix used as restraints (Figure 2D and Supplementary Figure S2). Taken together, this analysis shows that it is possible to consistently recover most of the 3D organization of a region of interest with 10 captured loci per Mb and with just 2–3% of all possible interactions within a region captured.

### Cell-type-specific organization of the β-globin locus

To illustrate the utility of our integrative approach in unveiling the differential organization of loci, we applied it to the genomic region surrounding the β-globin locus in three different cell-types (cb-Ery, nCD4 and Mon; ‘Materials and Methods’ section) for which pcHi-C data are available (33). The selected genomic region contains five coding genes (HBB, HBD, HBG1, HBG2 and HBE1) with developmental-stage-dependent expression (51), which is finely regulated by a set of upstream enhancers known as the locus control region (LCR) (52). This locus is known to be in an active conformation in cb-Ery, where the LCR is interacting mainly with expressed genes as HBB and HBD, but not in nCD4 and Mon cells (33).

**Figure 3.**
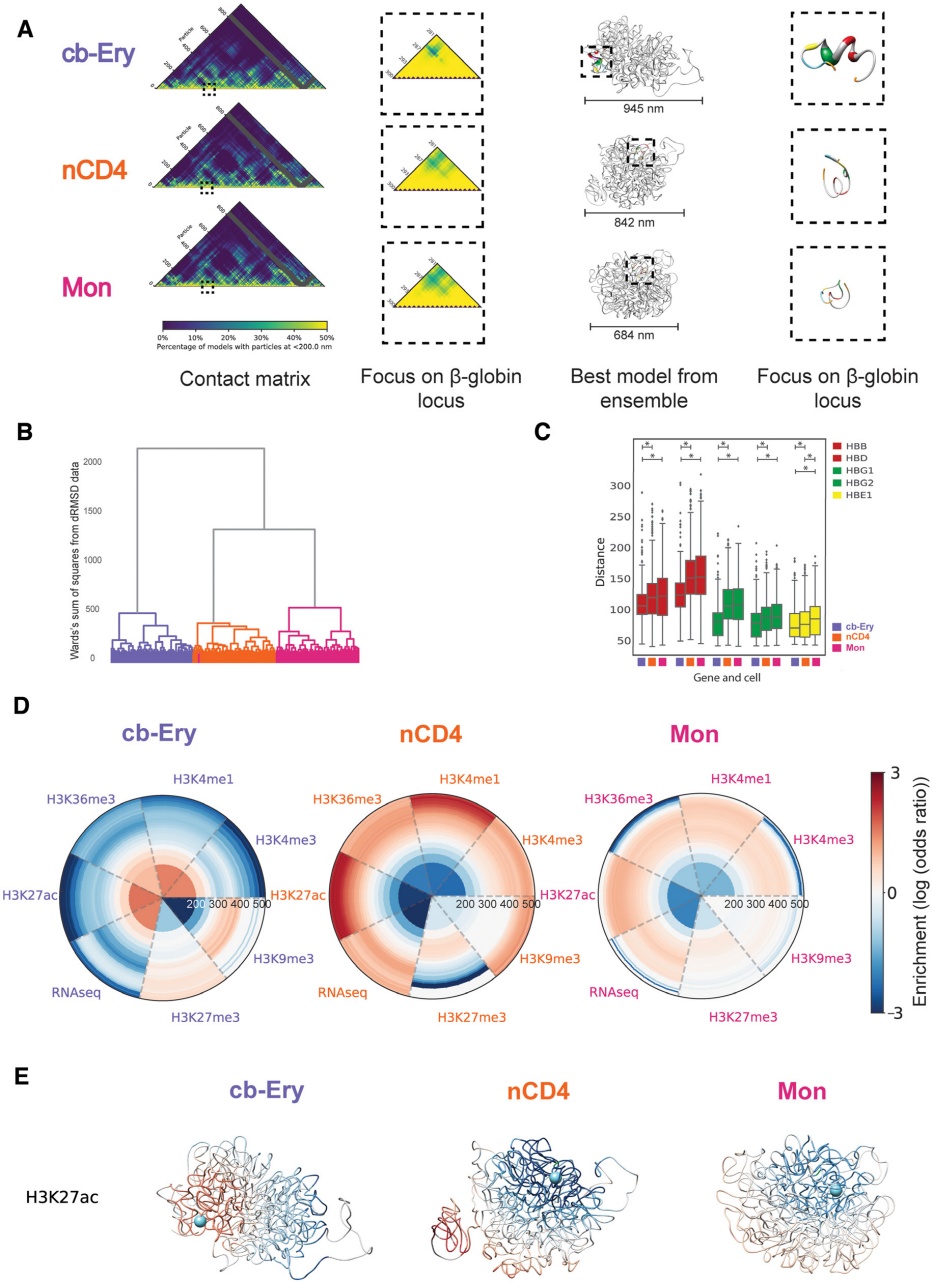
Cell-type-specific organization patterns of the β-globin locus. (**A**) β-globin locus in cb-Ery, nCD4 and Mon cell-types. From left to right: representation of the contact matrix derived from each of the model ensembles colour-coded from low (blue) to high (yellow) contact frequency (columns filtered due to low interaction data are coloured grey); zoom in of the β-globin locus in the matrix; best model from ensemble as assessed by the scoring function; zoom up of the β-globin locus in the model. Models are represented as a tube with thickness proportional to the cell-type expression profile (‘Materials and Methods’ section), the regulatory elements and genes in the β-globin locus are coloured as follow: HBB and HBD in red, HBG1 and HBG2 in green, HBE1 in yellow, LCR in blue and 3’HS1 and HS5 in orange. Model images were rendered with the Chimera visualization software (74). (**B**) Clustering tree (see ‘Hierarchical clustering of ensembles of 3D models’ section in Chromatin ensemble 3D analysis) of cb-Ery (purple), nCD4 (orange) and Mon (pink) model ensembles. (**C**) Cell-type-specific distance distributions between the particle containing HS3 site of the LCR and the β-globin genes (HBB, HBD, HBG1, HBG2, and HBE1, colour coded as in (A)) as observed in the ensemble of models. Box boundaries represent first and third quartiles, middle line represents median, and whiskers extend to 1.5 times the interquartile range (two-samples Kolmogorov–Smirnov test, asterisk indicate *P* < 0.007). (**D**) Radial plot showing the 3D enrichment around HS3 (‘Materials and Methods’ section). Each circumference shows the enrichment or depletion of features around HS3 on layers (up to 560 nm away from HS3) of non-overlapping volumes equal to the one of the initial sphere with radius of 200 nm. The colour bar shows the colour coding from highly depleted (blue) to highly enriched (red) features. (**E**) The representative 3D model of each of the ensembles (cb-Ery, Mon and nCD4) is represented as a tube and colour-coded by the 3D enrichment analysis of H3K27ac (from highly depleted in blue to highly enriched in red) around HS3 (represented as a light blue sphere).

First, we defined the optimal region to be modelled based on the interaction networks (in all cell-types) of the embryonic (HBG1 and HBG2) and adult (HBB and HBD) globin genes with the rest of the genome at 5 kb resolution (‘Materials and Methods’ section). The defined region spanned 4.7 Mb of chr11 (chr11:3 795 000–8 505 000 base-pairs (bp)) comprising several neighbouring genes and multiple long-range regulatory elements. By applying our integrative approach, we generated an ensemble of 1000 3D models for each cell-type. The packing of the genomic region was significantly different in each cell-types with median radius of gyration of 248 ± 3, 242 ± 2 and 237 ± 2 nm for cb-Ery, nCD4 and Mon, respectively (*P*-values < 9.1e^−163^ in each of the pairwise comparisons using two-samples Kolmogorov–Smirnov statistics) (Supplementary Figure S3A), with the topology of the region in cb-Ery being less tightly packed than in nCD4 and Mon. Each ensemble was then clustered by structural similarity (27) and the models from the most populated cluster were selected for the comparative analysis between cell-types. Clustering by dRMSD, confirmed that the topology of the region was markedly different in the three cell-types, with nCD4 and Mon folds being more similar between each other than with cb-Ery (Figure 3B). Particularly interesting is how the topology of the β-globin locus (chr11:5 201 270–5 302 470) varied in the three cell-types. Indeed, in Erythroblasts the β-globin locus appeared to be located further from the main core of the region as compared with naïve CD4+ T cells and Monocytes, with median distances between the centre of mass of the β-globin locus of 286, 243 and 207 nm in cb-Ery, nCD4 and Mon, respectively (*P*-values < 3.46e^−101^ in all the pairwise cell-type comparisons; two-samples Kolmogorov–Smirnov statistic) (Supplementary Figure S3B).

To characterize this further, we focused specifically on the β-globin locus and quantified its spatial organization with respect to hypersensitive site 3 (HS3) in the LCR, which is forming an intricate network of interaction with the β-globin genes (53) and is required for their activation (54). In line with this evidence, in the 3D ensemble of models representing cb-Ery cells, HS3 was significantly closer to HBB, HBD, HBG1, HBG2 and HBE1 genes than in the 3D ensemble of models representing nCD4 and Mon (*P*-values < 0.007, two-samples Kolmogorov–Smirnov test). In the latter two cell-types HS3 had a similar distance distribution with HBB, HBD, HBG1 and HBG2 genes (*P*-values > 0.01, two samples Kolmogorov–Smirnov test) (Figure 3C).

Performing 3D enrichment analysis of varied epigenetic features and expression levels around HS3 (‘Materials and Methods’ section), we unveiled a stark enrichment of active chromatin marks (H3K27ac, H3K36me, H3K4me1 and H3K4me3) and expression levels, and a clear depletion of inactive marks (H3K9me3 and H3K27me3) in cb-Ery. This 3D functional signature could not be inferred from the 2D genomic track (Supplementary Figure S4A) and was absent in nCD4 and Mon, where active chromatin marks and transcript levels were depleted (Figure 3D and E; Supplementary Figure S5). Overall, our models recapitulated the different 3D organization of the β-globin locus and highlight the existence of a specific 3D functional signature enriched in active chromatin features that characterized the active β-globin locus in cb-Ery.

### Active gene communities in cb-Ery: a cell-type-specific 3D signature

**Figure 4.**
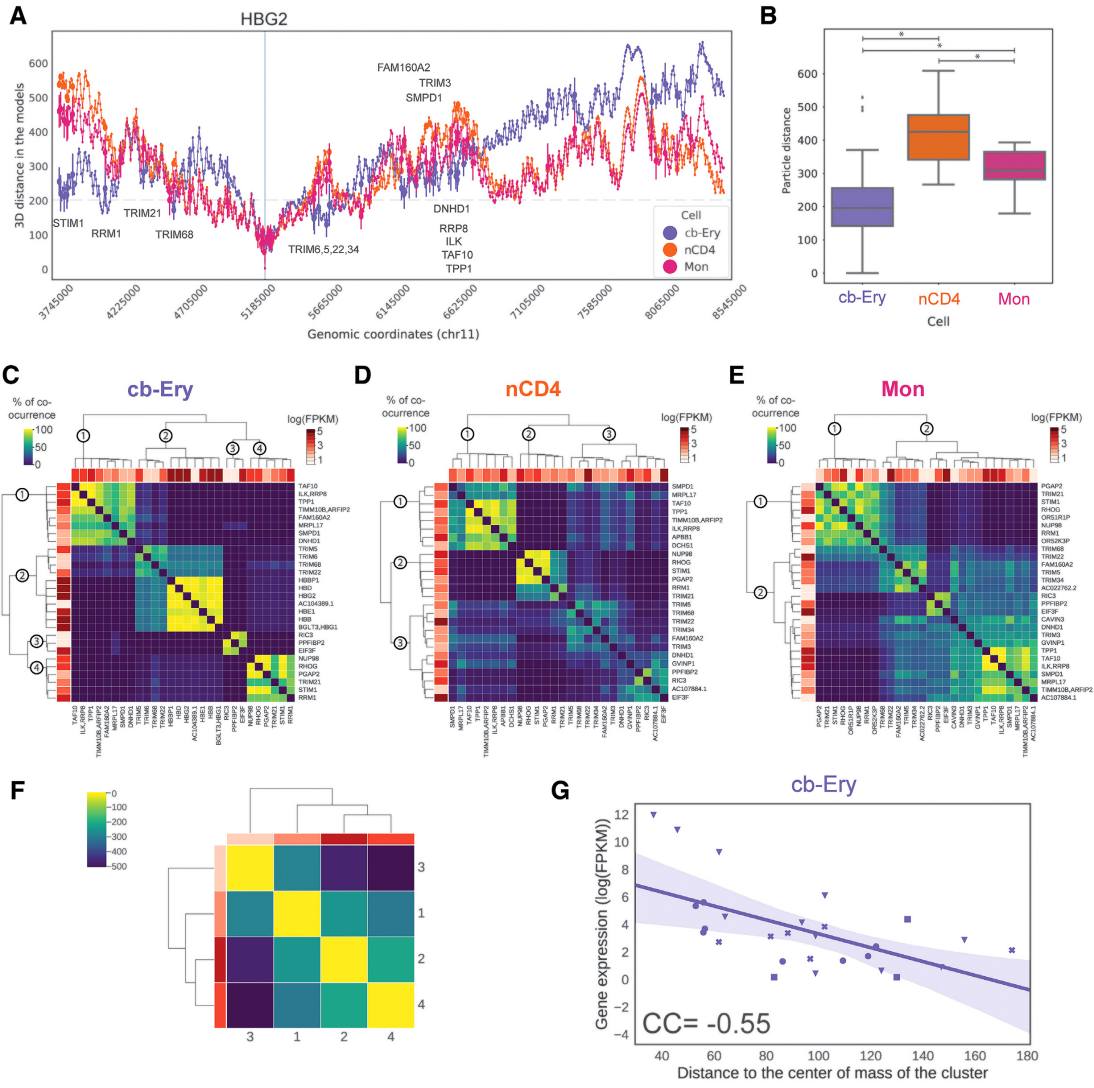
Communities of active genes as a cell-type-specific 3D signature in cb-Ery. (**A**) Line plot of the mean distances between the TSS of HBG2 (focus point, blue vertical line) and all other particles in the genomic region (chr11:3 795 000–8 504 999 bp) for cb-Ery (purple), nCD4 (orange) and Mon (pink) as calculated in each model ensembles. Error bar, indicating one standard deviation, is displayed for particles enclosing a transcribed gene (in at least one cell). The grey dashed line indicates 200 nm cut-off used in the analysis (‘Materials and Methods’ section). (**B**) Cell-type-specific distance distribution between particles enclosing the HBG2 gene and all transcribed genes in the genomic region (chr11:3 795 000–8 504 999 bp) for cb-Ery (purple), nCD4 (orange), and Mon (pink) as calculated in each model ensembles. Box boundaries represent first and third quartiles, middle line represents median, and whiskers extend to 1.5 times the interquartile range (two-samples Kolmogorov–Smirnov test, asterisk indicate *P*-values < 7.5e^−6^). (**C**–**E**) Hierarchical clustering of each genes based on the co-occurrence analysis (‘Materials and Methods’ section) in cb-Ery (C), nCD4 (D), and Mon (E). Co-occurrence value range from 0 (low, dark blue) to 100 (high, bright yellow). In each hierarchical tree the communities are labelled at their root branch. Per each gene the relative expression (log(FPKM) is shown in a scale of reds from 0 to 5. (**F**) Hierarchical clustering of the distances between the communities defined in cb-Ery (‘Materials and Methods’ section). Distance values are coloured in the matrix from dark blue to bright yellow and the average expression in log(FPKM) per community is coloured by ranking from lowest (lightest) to highest (darkest) in three different shades of red. (**G**) Relationship between gene expression in log(FPKM) and the median distance of the gene particles to the centre of mass of its own community in cb-Ery ensemble of models (‘Materials and Methods’ section). Purple line denotes the linear regression fit, the shading around the regression line represents the confidence interval, each community is represented with different symbols (circle community 1; inverse triangle community 2; square community 3; and ex community 4).

To examine whether the specific 3D functional signature of the active β-globin locus influence its genomic neighbourhood, we investigated its long-range interaction patterns. Comparative analysis of the distance profile between HBG2 (the most expressed gene in cb-Ery) and each of the selected loci (chr11: 3 795 000–8 505 000 bp), revealed the existence of an intricate cell-type-specific network of spatially proximal expressed genes (Figure 4A), in line with previous observations of transcribed genes co-localizing in space (24,55,56,57,58). This network comprised distal transcribed sites (even located at 1.4 Mb away as STIM1) that showed cell-type-specific spatial proximity. Indeed, HBG2 in cb-Ery was in closer proximity with all other expressed loci of the genomic neighbourhood than in nCD4 and Mon (Figure 4B).

To further characterize the cell-type-specific spatial distribution of these transcribed loci, we clustered their relative distances within the ensembles of 3D models and identified communities of expressed genomic loci (Figure 4C–E and ‘Materials and Methods’ section). Then, we quantified the amount of times a given community of expressed genomic loci occurred within the ensembles of 3D models (i.e. the co-occurrence score, ‘Materials and Methods’ section) and used this quantification as a proxy to define the ‘community stability’. This analysis revealed the existence of highly variable communities of expressed genomic loci that followed a cell-type-specific segregation in the 3D space. Interestingly, the organization of these communities was overall more stable in cb-Ery than in nCD4 and Mon, where less defined communities were identified. Indeed, as assessed by the mean inter-community co-occurrence scores (‘Materials and Methods’ section), the cb-Ery network was characterized by the presence of four stable communities (‘Materials and Methods’ section and Table 1). Whilst the nCD4 network was formed by three communities with overall low co-occurrence (although community 2 in this network showed a stability in line with the communities in the cb-Ery network), and the Mon network formed by only two unstable communities (‘Materials and Methods’ section and Table 1). Overall, the results highlight the presence of more defined 3D communities of expressed genes in cb-Ery as compared to nCD4 and Mon, suggesting that the co-occurrence of these segregated communities within an ensemble of possible folds is part of the cell-type-specific 3D signature.

**Table 1.** Communities stability assessment

Cell	Community	Mean inter-community co-occurrence	Average inter-community co-occurrence per cell
cb-Ery	1	2.96	3.06
	2	4.90	
	3	0.54	
	4	3.85	
nCD4	1	11.49	9.16
	2	3.83	
	3	12.17	
Mon	1	10.33	10.33
	2	10.33	

Description — Cell: the cell-type data used to reconstruct the chromatin; Community: the defined communities by Ward's clustering; Mean inter-community co-occurrence: communities stability score as defined in ‘Materials and Methods’ section; Average inter-community co-occurrence per cell: average mean inter-community co-occurrence value of all the communities in each of the cells.

Next, we investigated whether the stability of the 3D communities of expressed genes in cb-Ery could be related to the high levels of expression of the β-globin genes (highest as HBG2 with 10.86 FPKM, whilst the mean expression of all the other expressed genes in nCD4 and Mon was 2.45 and 2.10 FPKM, respectively). Clustering the distance distribution between the centres of mass of each community in cb-Ery (Figure 4F) revealed a clear hierarchical organization with the most expressed community, which included the highly expressed β-globin locus (Supplementary Table S3), located in the centre, and the least expressed community in the periphery. This pattern was not present in nCD4, and impossible to address in Mon with just two communities (Supplementary Figure S6A and B). This suggests a hierarchical organization in cb-Ery, in which the location in space of each of the communities and their levels of expression are related. Surprisingly, this hierarchy was also overall present at the community level in cb-Ery, where the distance between each gene to the centre of mass of the community and its expression were negatively correlated (CC: −0.55, *P*-value = 0.002; Figure 4G). This suggests the formation in cb-Ery of a gradient of expression within the community were the most expressed genes are located in the centre of their communities and the less expressed ones are preferentially located in the periphery in line with the organization previously observed for the alpha-globin locus (24). This overall community organization was not evident in nCD4 and Mon (Supplementary Figure S6C and D), thus suggesting that the high expression of the β-globin loci in cb-Ery could be associated with the establishment of a hierarchical organization in the loci.

## DISCUSSION

Here, we have introduced an integrative modelling method for the 3D reconstruction, analysis and interpretation of sparse 3C-based datasets such as pcHi-C. We also demonstrate its usability in the comparative 3D analysis of genomic regions using the β-globin locus as an example, showing that our method can detect cell-type-specific 3D organizational features within genomic regions that can lead to several important implications on the relationship between genomic function and spatial genome organization, such as the expression dependent organization of active loci.

Generally, the analysis and interpretation of sparse 3C-datasets is not trivial and specialized analytical tools are required. In the case of pcHi-C, the available tools (ChiCMaxima, Chicago, Chicdiff, Gothic, HiCapTools (59,60,61,62,63)) are mainly focused on the implementation of normalization strategies to reduce the impact of non-biological biases and on strategies to detect interaction between captured loci. Conversely, the integrative modelling method presented in this study has been designed for the analysis and interpretation of sparse 3C-datasets in their third dimension, allowing for data normalization, detection of significative interaction, and most importantly, the recovery of the full structural organization of a genomic region despite of the data sparseness.

Indeed, here we extensively tested our procedure by comparing models reconstructed directly from sparse and dense datasets, showing that 3D models reconstructed by the integrative modelling method for sparse data modelling are a good representation of the dense experiment. In fact, model reconstruction is only minorly affected by the intrinsic experimental biases of the capture experiment. Additionally, and most importantly, our model procedure reproduces remarkably well the whole 3D organization of the selected genomic regions even recovering the organization of loci that are not included as input restraints and are not readily observable in the sparse experiment.

Next, to assess whether the 3D reconstructed models were not only a *bona fide* representation of models based on Hi-C datasets, we used a ‘synthetic’ toy genome with known 3D organization (37) and proved that we can efficiently model sparse pcHi-C-like datasets using as few as 2–3% of all possible interaction data. Importantly, this quantification highlights how the degree of sparseness of the data is related to the efficiency of the 3D reconstruction process and provide a general guideline for sparse data modelling. In light of this, we speculate that our integrative approach could easily be applied to different type of 3C datasets with similar sparseness. For example, protein-centric chromatin conformation method such as HiChIP (19) could be used as input experiment to reconstruct the chromatin folding, assuming that the protein-capture biases of this type of experiments are similar to the promoter-capture biases observed in the pcHiC experiments.

Finally, to illustrate the utility of our integrative approach, we applied it to the β-globin locus, whose 3D organization has been extensively studied (51,53,64,65,66). We investigated this locus in three different cell-types (cb-Ery, nCD4 and Mon) and performed a comparative analysis between them. In agreement with previous studies (33), our models show that the topology of the β-globin locus varies in the three cell-types owing to their differential expression. Interestingly, our models also unveil that the globin HBG2 gene is embedded in an epigenetically active and highly transcribed neighbourhood in cb-Ery giving rise to a locus-specific 3D functional signature. This functional signature is absent in the models of other cell-types (nCD4 and Mon), where the locus is not expressed. We also show that this cell-specific organization, not only occurs proximally to the β-globin genes but also involves loci located at longer genomic distances (more than 1 Mb away). Indeed, our 3D comparative analysis unveiled the existence of an intricate cell-type-specific network of spatially proximal expressed genes that forms gene communities that are segregated in the 3D space in a cell-type-specific fashion. The identified communities are compatible with the formation of chromatin foci in which transcribed genes co-localize as a general mechanism to organize gene transcription (24,55,56,57,58,67). Interestingly, we observed that the co-occurrence within the ensemble of models of the identified cell-type-specific communities is cell-type dependent, with the cb-Ery communities network formed by more persistent communities than the nCD4 and Mon community networks. This suggests that also the degree of co-occurrence of the communities within the ensemble is an important feature for the identification of a cell-type-specific 3D signature. Additionally, we observed that in cb-Ery, where the β-globin genes are highly expressed, the communities present an overall hierarchical spatial organization, both between and within communities. This topology is dependent on the level of transcription with highly expressed entities (entire community or specific gene within a community) located in the core of the hierarchical 3D organization and low expressed entities found at the periphery. We hypothesize that the observed communities could represent cell-type-specific transcription factories (24,67,68,69) or phase-separated foci (70,71,72) organized following a gradient of transcription with high concentration of nascent transcripts and macromolecular protein complexes in the core of the assemblies that create a ‘sticky’ environment for the less expressed peripheral loci. This hierarchical organization is only marginally present in nCD4 and Mon, suggesting that it contributes to the cell-type-specific 3D signature characterizing the β-globin region in cb-Ery. However, the long-range interactions between the active β-globin locus and other active gene loci have been seen to be not dependent on the process of ongoing transcription or on the binding of RNAPII to regulatory elements (73), suggesting that the observed communities’ organization is more likely dependent on high concentrations of other macromolecular protein complexes in the ‘sticky’ core of the hierarchical 3D organization.

In summary, we have shown that sparse datasets like pcHi-C can be effectively used to model in 3D the spatial conformation of genomic domains. The resulting models retain most of the genomic region organization and recover also the organization of loci that are not readily observable in the sparse experiment. Importantly, this is achievable with a very small percentage (∼2–3%) of all possible interaction data in the genomic region. Additionally, our study not only provides a novel approach for sparse-data 3D modelling but also introduces new tools for the comparative analysis of genomic regions. Thus, it will aid the discovery of cell-type-specific 3D signatures and help deciphering complex mechanism underlying the cell-type-specific 3D genome organization.

## DATA AVAILABILITY

Hi-C data for GM12878 cell line were obtained from Gene Expression Omnibus (GEO) at the accession number GSE63525. pcHi-C data from GM12878 cell line were obtained from ArrayExpress at the accession number E-MTAB-2323. pcHi-C data for cb-Ery, nCD4 and Mon cells were obtained from the European Genome-phenome Archive at the accession number EGAS00001001911. Expression matrix for cb-Ery, nCD4 and Mon cells was downloaded from https://osf.io/u8tzp/ (GeneExpressionMatrix.txt.gz).

The code used to reconstruct 3D models from the sparse-data modelling approach, to analyze data and to generate figures is available in the GitHub repository (https://github.com/3DGenomes/SparseDataModelling).

## Supplementary Material

lqab017_Supplemental_File

## References

[1] Lieberman-Aiden E. , van Berkum N.L., Williams L., Imakaev M., Ragoczy T., Telling A., Amit I., Lajoie B.R., Sabo P.J., Dorschner M.O. et al. Comprehensive mapping of long-range interactions reveals folding principles of the human genome. Science. 2009; 326:289–293. 19815776 10.1126/science.1181369 PMC2858594

[2] Dixon J.R. , Selvaraj S., Yue F., Kim A., Li Y., Shen Y., Hu M., Liu J.S., Ren B. Topological domains in mammalian genomes identified by analysis of chromatin interactions. Nature. 2012; 485:376–380. 22495300 10.1038/nature11082 PMC3356448

[3] Nora E.P. , Lajoie B.R., Schulz E.G., Giorgetti L., Okamoto I., Servant N., Piolot T., van Berkum N.L., Meisig J., Sedat J. et al. Spatial partitioning of the regulatory landscape of the X-inactivation centre. Nature. 2012; 485:381–385. 22495304 10.1038/nature11049 PMC3555144

[4] Sexton T. , Yaffe E., Kenigsberg E., Bantignies F., Leblanc B., Hoichman M., Parrinello H., Tanay A., Cavalli G. Three-dimensional folding and functional organization principles of the *Drosophila* genome. Cell. 2012; 148:458–472. 22265598 10.1016/j.cell.2012.01.010

[5] Hsieh T.S. , Cattoglio C., Slobodyanyuk E., Hansen A.S., Rando O.J., Tjian R., Darzacq X. Resolving the 3D landscape of transcription-linked mammalian chromatin folding. Mol. Cell. 2020; 78:539–553. 32213323 10.1016/j.molcel.2020.03.002 PMC7703524

[6] Rao S.S. , Huntley M.H., Durand N.C., Stamenova E.K., Bochkov I.D., Robinson J.T., Sanborn A.L., Machol I., Omer A.D., Lander E.S. et al. A 3D map of the human genome at kilobase resolution reveals principles of chromatin looping. Cell. 2014; 159:1665–1680. 25497547 10.1016/j.cell.2014.11.021 PMC5635824

[7] Bonev B. , Mendelson Cohen N., Szabo Q., Fritsch L., Papadopoulos G.L., Lubling Y., Xu X., Lv X., Hugnot J.P., Tanay A. et al. Multiscale 3D genome rewiring during mouse neural development. Cell. 2017; 171:557–572. 29053968 10.1016/j.cell.2017.09.043 PMC5651218

[8] Zheng H. , Xie W. The role of 3D genome organization in development and cell differentiation. Nat. Rev. Mol. Cell Biol. 2019; 20:535–550. 31197269 10.1038/s41580-019-0132-4

[9] Kempfer R. , Pombo A. Methods for mapping 3D chromosome architecture. Nat. Rev. Genet. 2020; 21:207–226. 31848476 10.1038/s41576-019-0195-2

[10] Dekker J. , Marti-Renom M.A., Mirny L.A. Exploring the three-dimensional organization of genomes: interpreting chromatin interaction data. Nat. Rev. Genet. 2013; 14:390–403. 23657480 10.1038/nrg3454 PMC3874835

[11] Nagano T. , Lubling Y., Stevens T.J., Schoenfelder S., Yaffe E., Dean W., Laue E.D., Tanay A., Fraser P. Single-cell Hi-C reveals cell-to-cell variability in chromosome structure. Nature. 2013; 502:59–64. 24067610 10.1038/nature12593 PMC3869051

[12] Ramani V. , Deng X., Qiu R., Lee C., Disteche C.M., Noble W.S., Shendure J., Duan Z. Sci-Hi-C: a single-cell Hi-C method for mapping 3D genome organization in large number of single cells. Methods. 2020; 170:61–68. 31536770 10.1016/j.ymeth.2019.09.012 PMC6949367

[13] Flyamer I.M. , Gassler J., Imakaev M., Brandao H.B., Ulianov S.V., Abdennur N., Razin S.V., Mirny L.A., Tachibana-Konwalski K. Single-nucleus Hi-C reveals unique chromatin reorganization at oocyte-to-zygote transition. Nature. 2017; 544:110–114. 28355183 10.1038/nature21711 PMC5639698

[14] Hsieh T.H. , Weiner A., Lajoie B., Dekker J., Friedman N., Rando O.J. Mapping Nucleosome Resolution Chromosome Folding in Yeast by Micro-C. Cell. 2015; 162:108–119. 26119342 10.1016/j.cell.2015.05.048 PMC4509605

[15] Beagrie R.A. , Scialdone A., Schueler M., Kraemer D.C., Chotalia M., Xie S.Q., Barbieri M., de Santiago I., Lavitas L.M., Branco M.R. et al. Complex multi-enhancer contacts captured by genome architecture mapping. Nature. 2017; 543:519–524. 28273065 10.1038/nature21411 PMC5366070

[16] Quinodoz S.A. , Ollikainen N., Tabak B., Palla A., Schmidt J.M., Detmar E., Lai M.M., Shishkin A.A., Bhat P., Takei Y. et al. Higher-order inter-chromosomal hubs shape 3D genome organization in the nucleus. Cell. 2018; 174:744–757. 29887377 10.1016/j.cell.2018.05.024 PMC6548320

[17] van de Werken H.J. , de Vree P.J., Splinter E., Holwerda S.J., Klous P., de Wit E., de Laat W. 4C technology: protocols and data analysis. Methods Enzymol. 2012; 513:89–112. 22929766 10.1016/B978-0-12-391938-0.00004-5

[18] Allahyar A. , Vermeulen C., Bouwman B.A.M., Krijger P.H.L., Verstegen M., Geeven G., van Kranenburg M., Pieterse M., Straver R., Haarhuis J.H.I. et al. Enhancer hubs and loop collisions identified from single-allele topologies. Nat. Genet. 2018; 50:1151–1160. 29988121 10.1038/s41588-018-0161-5

[19] Mumbach M.R. , Rubin A.J., Flynn R.A., Dai C., Khavari P.A., Greenleaf W.J., Chang H.Y. HiChIP: efficient and sensitive analysis of protein-directed genome architecture. Nat. Methods. 2016; 13:919–922. 27643841 10.1038/nmeth.3999 PMC5501173

[20] Schoenfelder S. , Furlan-Magaril M., Mifsud B., Tavares-Cadete F., Sugar R., Javierre B.M., Nagano T., Katsman Y., Sakthidevi M., Wingett S.W. et al. The pluripotent regulatory circuitry connecting promoters to their long-range interacting elements. Genome Res. 2015; 25:582–597. 25752748 10.1101/gr.185272.114 PMC4381529

[21] Bendandi A. , Dante S., Zia S.R., Diaspro A., Rocchia W. Chromatin compaction multiscale modeling: a complex synergy between theory, simulation, and experiment. Front. Mol. Biosci. 2020; 7:15–21. 32158765 10.3389/fmolb.2020.00015 PMC7051991

[22] Oluwadare O. , Highsmith M., Cheng J. An overview of methods for reconstructing 3-D chromosome and genome structures from Hi-C data. Biol. Proc. Online. 2019; 21:7. 10.1186/s12575-019-0094-0 PMC6482566 31049033

[23] Serra F. , Di Stefano M., Spill Y.G., Cuartero Y., Goodstadt M., Bau D., Marti-Renom M.A. Restraint-based three-dimensional modeling of genomes and genomic domains. FEBS Lett. 2015; 589:2987–2995. 25980604 10.1016/j.febslet.2015.05.012

[24] Baù D. , Sanyal A., Lajoie B.R., Capriotti E., Byron M., Lawrence J.B., Dekker J., Marti-Renom M.A. The three-dimensional folding of the alpha-globin gene domain reveals formation of chromatin globules. Nat. Struct. Mol. Biol. 2011; 18:107–114. 21131981 10.1038/nsmb.1936 PMC3056208

[25] Tjong H. , Li W., Kalhor R., Dai C., Hao S., Gong K., Zhou Y., Li H., Zhou X.J., Le Gros M.A. et al. Population-based 3D genome structure analysis reveals driving forces in spatial genome organization. Proc. Natl Acad. Sci. U.S.A. 2016; 113:E1663–E1672. 26951677 10.1073/pnas.1512577113 PMC4812752

[26] Hua N. , Tjong H., Shin H., Gong K., Zhou X.J., Alber F. Producing genome structure populations with the dynamic and automated PGS software. Nat. Protoc. 2018; 13:915–926. 29622804 10.1038/nprot.2018.008 PMC6043163

[27] Serra F. , Bau D., Goodstadt M., Castillo D., Filion G.J., Marti-Renom M.A. Automatic analysis and 3D-modelling of Hi-C data using TADbit reveals structural features of the fly chromatin colors. PLoS Comput. Biol. 2017; 13:e1005665. 28723903 10.1371/journal.pcbi.1005665 PMC5540598

[28] Irastorza-Azcarate I. , Acemel R.D., Tena J.J., Maeso I., Gomez-Skarmeta J.L., Devos D.P. 4Cin: a computational pipeline for 3D genome modeling and virtual Hi-C analyses from 4C data. PLoS Comput. Biol. 2018; 14:e1006030. 29522512 10.1371/journal.pcbi.1006030 PMC5862518

[29] Di Stefano M. , Stadhouders R., Farabella I., Castillo D., Serra F., Graf T., Marti-Renom M.A. Transcriptional activation during cell reprogramming correlates with the formation of 3D open chromatin hubs. Nat. Commun. 2020; 11:2564. 32444798 10.1038/s41467-020-16396-1 PMC7244774

[30] Paulsen J. , Gramstad O., Collas P. Manifold based optimization for single-cell 3D genome reconstruction. PLoS Comput. Biol. 2015; 11:e1004396. 26262780 10.1371/journal.pcbi.1004396 PMC4532452

[31] Stevens T.J. , Lando D., Basu S., Atkinson L.P., Cao Y., Lee S.F., Leeb M., Wohlfahrt K.J., Boucher W., O'Shaughnessy-Kirwan A. et al. 3D structures of individual mammalian genomes studied by single-cell Hi-C. Nature. 2017; 544:59–64. 28289288 10.1038/nature21429 PMC5385134

[32] Mifsud B. , Tavares-Cadete F., Young A.N., Sugar R., Schoenfelder S., Ferreira L., Wingett S.W., Andrews S., Grey W., Ewels P.A. et al. Mapping long-range promoter contacts in human cells with high-resolution capture Hi-C. Nat. Genet. 2015; 47:598–606. 25938943 10.1038/ng.3286

[33] Javierre B.M. , Burren O.S., Wilder S.P., Kreuzhuber R., Hill S.M., Sewitz S., Cairns J., Wingett S.W., Varnai C., Thiecke M.J. et al. Lineage-specific genome architecture links enhancers and non-coding disease variants to target gene promoters. Cell. 2016; 167:1369–1384. 27863249 10.1016/j.cell.2016.09.037 PMC5123897

[34] Vidal E. , le Dily F., Quilez J., Stadhouders R., Cuartero Y., Graf T., Marti-Renom M.A., Beato M., Filion G.J. OneD: increasing reproducibility of Hi-C samples with abnormal karyotypes. Nucleic Acids Res. 2018; 46:e49. 29394371 10.1093/nar/gky064 PMC5934634

[35] Yang T. , Zhang F., Yardimci G.G., Song F., Hardison R.C., Noble W.S., Yue F., Li Q. HiCRep: assessing the reproducibility of Hi-C data using a stratum-adjusted correlation coefficient. Genome Res. 2017; 27:1939–1949. 28855260 10.1101/gr.220640.117 PMC5668950

[36] Imakaev M. , Fudenberg G., McCord R.P., Naumova N., Goloborodko A., Lajoie B.R., Dekker J., Mirny L.A. Iterative correction of Hi-C data reveals hallmarks of chromosome organization. Nat. Methods. 2012; 9:999–1003. 22941365 10.1038/nmeth.2148 PMC3816492

[37] Trussart M. , Serra F., Bau D., Junier I., Serrano L., Marti-Renom M.A. Assessing the limits of restraint-based 3D modeling of genomes and genomic domains. Nucleic Acids Res. 2015; 43:3465–3477. 25800747 10.1093/nar/gkv221 PMC4402535

[38] Di Stefano M. , Rosa A., Belcastro V., di Bernardo D., Micheletti C. Colocalization of coregulated genes: a steered molecular dynamics study of human chromosome 19. PLoS Comput. Biol. 2013; 9:e1003019. 23555238 10.1371/journal.pcbi.1003019 PMC3610629

[39] Kremer K. , Grest G.S. Dynamics of entangled linear polymer melts: a molecular-dynamics simulation. J. Chem. Phys. 1990; 92:5057–5086.

[40] Rosa A. , Everaers R. Structure and dynamics of interphase chromosomes. PLoS Comput. Biol. 2008; 4:e1000153. 18725929 10.1371/journal.pcbi.1000153 PMC2515109

[41] Polak E. , Ribiere G. Note sur la convergence de méthodes de directions conjuguées. Rev. Fran Inf. Rech. Op. 1969; 16:35–43.

[42] Virtanen P. , Gommers R., Oliphant T.E., Haberland M., Reddy T., Cournapeau D., Burovski E., Peterson P., Weckesser W., Bright J. et al. SciPy 1.0: fundamental algorithms for scientific computing in Python. Nat. Methods. 2020; 17:261–272. 32015543 10.1038/s41592-019-0686-2 PMC7056644

[43] Zwillinger D. , Kokoska S. RC Standard Probability and Statistics Tables and Formulae. 2000; Boca Raton, FL Chapman & Hall/CRC.

[44] Ward J.H. Hierarchical grouping to optimize an objective function. J. Am. Statist. Assoc. 1963; 58:236–244.

[45] Smedley D. , Haider S., Durinck S., Pandini L., Provero P., Allen J., Arnaiz O., Awedh M.H., Baldock R., Barbiera G. et al. The BioMart community portal: an innovative alternative to large, centralized data repositories. Nucleic Acids Res. 2015; 43:W589–W598. 25897122 10.1093/nar/gkv350 PMC4489294

[46] Caliński T. , Harabasz J. A dendrite method for cluster analysis. Commun. Stat. 1974; 3:1–27.

[47] Pedregosa F. , Varoquaux G., Gramfort A., Michel V., Thirion B., Grisel O., Blondel M., Prettenhofer P., Weiss R., Dubourg V. et al. Scikit-learn: machine learning in Python. J. Mach. Learn. Res. 2011; 12:2825–2830.

[48] Jung I. , Schmitt A., Diao Y., Lee A.J., Liu T., Yang D., Tan C., Eom J., Chan M., Chee S. et al. A compendium of promoter-centered long-range chromatin interactions in the human genome. Nat. Genet. 2019; 51:1442–1449. 31501517 10.1038/s41588-019-0494-8 PMC6778519

[49] Miguel-Escalada I. , Bonas-Guarch S., Cebola I., Ponsa-Cobas J., Mendieta-Esteban J., Atla G., Javierre B.M., Rolando D.M.Y., Farabella I., Morgan C.C. et al. Human pancreatic islet three-dimensional chromatin architecture provides insights into the genetics of type 2 diabetes. Nat. Genet. 2019; 51:1137–1148. 31253982 10.1038/s41588-019-0457-0 PMC6640048

[50] Russel D. , Lasker K., Webb B., Velazquez-Muriel J., Tjioe E., Schneidman-Duhovny D., Peterson B., Sali A. Putting the pieces together: integrative modeling platform software for structure determination of macromolecular assemblies. PLoS Biol. 2012; 10:e1001244. 22272186 10.1371/journal.pbio.1001244 PMC3260315

[51] Palstra R.J. , Tolhuis B., Splinter E., Nijmeijer R., Grosveld F., de Laat W. The beta-globin nuclear compartment in development and erythroid differentiation. Nat. Genet. 2003; 35:190–194. 14517543 10.1038/ng1244

[52] Levings P.P. , Bungert J. The human beta-globin locus control region. Eur. J. Biochem. 2002; 269:1589–1599. 11895428 10.1046/j.1432-1327.2002.02797.x

[53] Liu X. , Zhang Y., Chen Y., Li M., Zhou F., Li K., Cao H., Ni M., Liu Y., Gu Z. et al. In situ capture of chromatin interactions by biotinylated dCas9. Cell. 2017; 170:1028–1043. 28841410 10.1016/j.cell.2017.08.003 PMC6857456

[54] Fraser P. , Pruzina S., Antoniou M., Grosveld F. Each hypersensitive site of the human beta-globin locus control region confers a different developmental pattern of expression on the globin genes. Genes Dev. 1993; 7:106–113. 8422981 10.1101/gad.7.1.106

[55] Fraser P. , Bickmore W. Nuclear organization of the genome and the potential for gene regulation. Nature. 2007; 447:413–417. 17522674 10.1038/nature05916

[56] Jackson D.A. , Hassan A.B., Errington R.J., Cook P.R. Visualization of focal sites of transcription within human nuclei. EMBO J. 1993; 12:1059–1065. 8458323 10.1002/j.1460-2075.1993.tb05747.x PMC413307

[57] Osborne C.S. , Chakalova L., Brown K.E., Carter D., Horton A., Debrand E., Goyenechea B., Mitchell J.A., Lopes S., Reik W. et al. Active genes dynamically colocalize to shared sites of ongoing transcription. Nat. Genet. 2004; 36:1065–1071. 15361872 10.1038/ng1423

[58] Osborne C.S. , Chakalova L., Mitchell J.A., Horton A., Wood A.L., Bolland D.J., Corcoran A.E., Fraser P. Myc dynamically and preferentially relocates to a transcription factory occupied by Igh. PLoS Biol. 2007; 5:e192. 17622196 10.1371/journal.pbio.0050192 PMC1945077

[59] Ben Zouari Y. , Molitor A.M., Sikorska N., Pancaldi V., Sexton T. ChiCMaxima: a robust and simple pipeline for detection and visualization of chromatin looping in Capture Hi-C. Genome Biol. 2019; 20:102. 31118054 10.1186/s13059-019-1706-3 PMC6532271

[60] Cairns J. , Freire-Pritchett P., Wingett S.W., Varnai C., Dimond A., Plagnol V., Zerbino D., Schoenfelder S., Javierre B.M., Osborne C. et al. CHiCAGO: robust detection of DNA looping interactions in Capture Hi-C data. Genome Biol. 2016; 17:127. 27306882 10.1186/s13059-016-0992-2 PMC4908757

[61] Cairns J. , Orchard W.R., Malysheva V., Spivakov M. Chicdiff: a computational pipeline for detecting differential chromosomal interactions in Capture Hi-C data. Bioinformatics. 2019; 35:4764–4766. 31197313 10.1093/bioinformatics/btz450 PMC6853696

[62] Mifsud B. , Martincorena I., Darbo E., Sugar R., Schoenfelder S., Fraser P., Luscombe N.M. GOTHiC, a probabilistic model to resolve complex biases and to identify real interactions in Hi-C data. PLoS One. 2017; 12:e0174744. 28379994 10.1371/journal.pone.0174744 PMC5381888

[63] Anil A. , Spalinskas R., Akerborg O., Sahlen P. HiCapTools: a software suite for probe design and proximity detection for targeted chromosome conformation capture applications. Bioinformatics. 2018; 34:675–677. 29444232 10.1093/bioinformatics/btx625 PMC6368139

[64] Brown J.M. , Leach J., Reittie J.E., Atzberger A., Lee-Prudhoe J., Wood W.G., Higgs D.R., Iborra F.J., Buckle V.J. Coregulated human globin genes are frequently in spatial proximity when active. J. Cell Biol. 2006; 172:177–187. 16418531 10.1083/jcb.200507073 PMC2063548

[65] Schubeler D. , Francastel C., Cimbora D.M., Reik A., Martin D.I., Groudine M. Nuclear localization and histone acetylation: a pathway for chromatin opening and transcriptional activation of the human beta-globin locus. Genes Dev. 2000; 14:940–950. 10783166 PMC316536

[66] Huang P. , Keller C.A., Giardine B., Grevet J.D., Davies J.O.J., Hughes J.R., Kurita R., Nakamura Y., Hardison R.C., Blobel G.A Comparative analysis of three-dimensional chromosomal architecture identifies a novel fetal hemoglobin regulatory element. Genes Dev. 2017; 31:1704–1713. 28916711 10.1101/gad.303461.117 PMC5647940

[67] Sanyal A. , Bau D., Marti-Renom M.A., Dekker J. Chromatin globules: a common motif of higher order chromosome structure?. Curr. Opin. Cell Biol. 2011; 23:325–331. 21489772 10.1016/j.ceb.2011.03.009 PMC3109114

[68] Sutherland H. , Bickmore W.A. Transcription factories: gene expression in unions?. Nat. Rev. Genet. 2009; 10:457–466. 19506577 10.1038/nrg2592

[69] Iborra F.J. , Pombo A., Jackson D.A., Cook P.R. Active RNA polymerases are localized within discrete transcription "factories' in human nuclei. J. Cell Sci. 1996; 109:1427–1436. 8799830 10.1242/jcs.109.6.1427

[70] Gurumurthy A. , Shen Y., Gunn E.M., Bungert J. Phase separation and transcription regulation: are super-enhancers and locus control regions primary sites of transcription complex assembly?. Bioessays. 2019; 41:e1800164. 30500078 10.1002/bies.201800164 PMC6484441

[71] Boija A. , Klein I.A., Sabari B.R., Dall’Agnese A., Coffey E.L., Zamudio A.V., Li C.H., Shrinivas K., Manteiga J.C., Hannett N.M. et al. Transcription factors activate genes through the phase-separation capacity of their activation domains. Cell. 2018; 175:1842–1855. 30449618 10.1016/j.cell.2018.10.042 PMC6295254

[72] Cho W.K. , Spille J.H., Hecht M., Lee C., Li C., Grube V., Cisse II. Mediator and RNA polymerase II clusters associate in transcription-dependent condensates. Science. 2018; 361:412–415. 29930094 10.1126/science.aar4199 PMC6543815

[73] Palstra R.J. , Simonis M., Klous P., Brasset E., Eijkelkamp B., de Laat W. Maintenance of long-range DNA interactions after inhibition of ongoing RNA polymerase II transcription. PLoS One. 2008; 3:e1661. 18286208 10.1371/journal.pone.0001661 PMC2243019

[74] Pettersen E.F. , Goddard T.D., Huang C.C., Couch G.S., Greenblatt D.M., Meng E.C., Ferrin T.E. UCSF chimera—a visualization system for exploratory research and analysis. J. Comput. Chem. 2004; 25:1605–1612. 15264254 10.1002/jcc.20084

